# Disruption of *SATB2* or its long-range *cis*-regulation by SOX9 causes a syndromic form of Pierre Robin sequence

**DOI:** 10.1093/hmg/ddt647

**Published:** 2013-12-20

**Authors:** Jacqueline K. Rainger, Shipra Bhatia, Hemant Bengani, Philippe Gautier, Joe Rainger, Matt Pearson, Morad Ansari, Jayne Crow, Felicity Mehendale, Bozena Palinkasova, Michael J. Dixon, Pamela J. Thompson, Mar Matarin, Sanjay M. Sisodiya, Dirk A. Kleinjan, David R. FitzPatrick

**Affiliations:** 1 MRC Human Genetics Unit, MRC Institute of Genetic and Molecular Medicine, University of Edinburgh, Edinburgh EH4 2XU, UK; 2 Adult Learning Disability Services, Lothian University Hospitals Trust, 65 Morningside Drive, Edinburgh EH10 5NQ, UK; 3 Cleft Lip and Palate Service, Royal Hospital for Sick Children, Edinburgh EH9 1LF, UK; 4 Faculty of Medical and Human Sciences, Michael Smith Building, University of Manchester, Oxford Road, Manchester M13 9PT, UK; 5 Department of Clinical and Experimental Epilepsy, National Hospital for Neurology and Neurosurgery, Queen Square, London WC1N 3BG, UK; 6 Epilepsy Society, Chalfont-St-Peter, BuckinghamshireSL9 0RJ, UK

## Abstract

Heterozygous loss-of-function (LOF) mutations in the gene encoding the DNA-binding protein, SATB2, result in micrognathia and cleft palate in both humans and mice. In three unrelated individuals, we show that translocation breakpoints (BPs) up to 896 kb 3′ of *SATB2* polyadenylation site cause a phenotype which is indistinguishable from that caused by *SATB2* LOF mutations. This syndrome comprises long nose, small mouth, micrognathia, cleft palate, arachnodactyly and intellectual disability. These BPs map to a gene desert between *PLCL1* and *SATB2.* We identified three putative *cis*-regulatory elements (CRE1–3) using a comparative genomic approach each of which would be placed in *trans* relative to *SATB2* by all three BPs. CRE1–3 each bind p300 and mono-methylated H3K4 consistent with enhancer function. *In silico* analysis suggested that CRE1–3 contain one or more conserved SOX9-binding sites, and this binding was confirmed using chromatin immunoprecipitation on cells derived from mouse embryonic pharyngeal arch. Interphase bacterial artificial chromosome fluorescence *in situ* hybridization measurements in embryonic craniofacial tissues showed that the orthologous region in mice exhibits *Satb2* expression-dependent chromatin decondensation consistent with *Satb2* being a target gene of CRE1–3. To assess their *in vivo* function, we made multiple stable reporter transgenic lines for each enhancer in zebrafish. CRE2 was shown to drive *SATB2*-like expression in the embryonic craniofacial region. This expression could be eliminated by mutating the *SOX9*-binding site of CRE2. These observations suggest that *SATB2* and *SOX9* may be acting together via complex *cis*-regulation to coordinate the growth of the developing jaw.

## INTRODUCTION

Pierre Robin sequence (PRS) is a clinically important subgroup of orofacial clefting defined by; micrognathia, *U*-shaped cleft palate, glossoptosis and obstructive apnea. The primary anomaly in PRS is considered to be failure of mandibular growth in the period prior to fusion of the embryonic palate resulting in abnormal placement of the tongue in the primitive oral cavity causing a physical obstruction to fusion of the posterior palatal shelves (1–3). A critical role for the collagen species COL2A1, COL11A1 and COL11A2 in the pathogenesis of PRS is suggested by the identification of heterozygous, dominant-negative, intragenic mutations in ∼30% of cases (i.e. Stickler syndrome-related PRS) (4–6). This role is further supported by the discovery that a proportion of non-syndromal PRS cases is caused by long-range *cis*-regulatory mutations (CRMs) within the large gene desert surrounding *SOX9* (7)*.* In the autosomal dominant families reported with these regulatory mutations affecting *SOX9*, PRS represents the most severe end of a spectrum that includes cleft palate with micrognathia and isolated micrognathia. A similar spectrum of severity is seen in Stickler and Stickler-like syndromes. It is notable that SOX9 is a direct transcriptional activator of *COL2A1* and *COL11A2* (8) suggesting that the growth failure of the jaw in both Stickler and SOX9-related PRS may have a shared pathogenesis via defects in the connective tissue matrix during development.

We previously reported two-unrelated cases with *de novo* apparently balanced chromosomal rearrangement (DNABCR) breakpoints (BPs) involving 2q33 associated with cleft palate, micrognathia, arachnodactyly, intellectual disability and a characteristic facial appearance (9). BP mapping implicated loss of function of *SATB2* as the cause of the clinical phenotype (10) with the transcription unit being directly disrupted in one case and the BP located 130 kb 3′ of the polyadenylation site within a large gene desert in the other. The latter case was hypothesized to represent a CRM. Support for a causative role for *SATB2* haploinsufficiency in craniofacial malformations was provided by patients with independent gene-disrupting DNABCRs (11,12), whole gene (13–15) and intragenic deletions (16) and a single nonsense mutation (17) associated with phenotypes that significantly overlap with the original cases.

SATB2 is a CUT and HOX domain-containing DNA-binding protein that shows site- and stage-specific expression during craniofacial and brain development. SATB2 differs in only 3 of 733 amino acids positions between human and mouse primary sequence. Human SATB2 is encoded by the *SATB2* gene that is located within chromosome band 2q33 (chr2:200 134 223–200 329 831 hg19). SATB2 is SUMOylated and associates with the nuclear matrix in pre-B cells (18). A non-redundant role for SATB2 in craniofacial development was confirmed when targeted inactivation of the gene in mouse embryos was shown to result in severe midline facial malformations in homozygous embryos with cleft palate also occurring in heterozygotes at lower penetrance (19). SATB2 also has an important role in specifying callosal (contralateral intracortical) projection of post-mitotic neurons in the developing cortex (20,21). *SATB2* and *SATB1*, the only close mammalian homolog, appear to be paralogs of a single invertebrate gene. In *Drosophila melanogaster*, this gene is known as defective proventriculus that is required for wing, midgut, leg joint, ommatidial and male accessory gland development (22–24).

In this paper, we update and extend the phenotypic characteristics of one of the original cases, re-interpret a case from the literature and report a further case with *de novo* balanced reciprocal translocation BP mapping to the gene desert adjacent to *SATB2* associated with PRS and a distinctive craniofacial appearance. We show that specific regions of this gene desert show *SATB2*-transcription-dependent chromatin de-condensation during craniofacial development. Furthermore, highly conserved non-coding elements upstream of this BP bind SOX9 and drive expression in transgenic animals consistent with them acting as *SATB2* enhancers. We also confirm and extend the clinical and radiological features associated with disruption of SATB2 function and suggest that this represents a clinically recognizable syndrome.

## RESULTS

### Potential CRM affecting SATB2

#### Case 1 46,XX,t(2;11)(q32;p14) *de novo*

**Figure 1.**
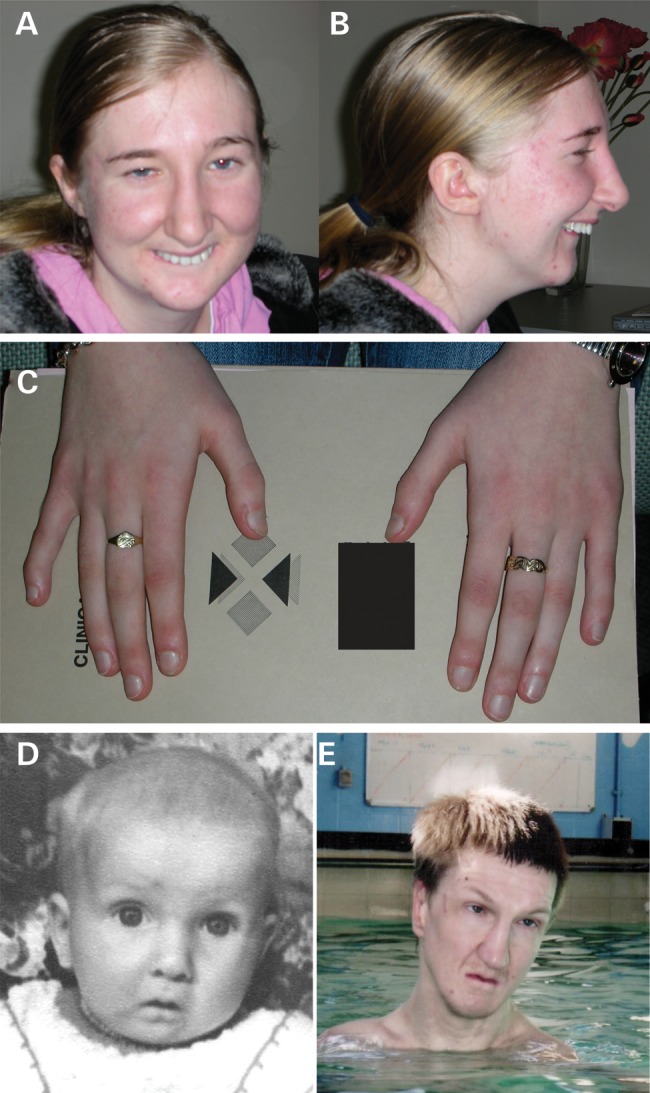
Clinical phenotype of SATB2-related PRS. Photographs of Case 1. Significant facial features include long nose, micrognathia and small mouth (**A** and **B**). Hands are notable for long slender fingers (**C**). Facial photographs of Case 2 at 10 weeks of age (**D**) and 31 years (**E**) showing small mouth (D and E), micrognathia (D) and long nose (E).

This individual was included in the original gene identification report (10). At that time, the 2q32 BP in this case was postulated to result in a CRM. She was clinically re-evaluated at the age of 24 years. Her early growth, development and general medical history are summarized in Table 1. She was noted to have had a long thin face with a prominent nasal bridge, a small mouth, micrognathia (Fig. 1A and B) and bilateral arachnodactyly (Fig. 1C). She has significant intellectual disability and requires constant supervision for reasons of her personal safety. These features were essentially unchanged from the original clinical report (9). She had a high-resolution magnetic resonance imaging (MRI) of her brain and detailed neuropsychology assessment at the age of 22. The MRI showed that the left lateral ventricle was larger than the right, with mild asymmetry of the cerebral hemispheres and cranium, without obvious malformation. The anterior commissure was small but present. The corpus callosum was present and unremarkable. Her intellectual level had previously been assessed to fall within the mild learning disability range IQ of ∼70–80. Her performance was impaired on measures of verbal learning and immediate visual recall (<1st centile). She benefited from rehearsal, and her delayed retention of both verbal and visual material was average (25–50th centile). Her verbal working memory and verbal fluency were weak (<1st centile). Her performance was impaired on a measure of response inhibition and a visuospatial apraxia was recorded. These findings imply widespread cognitive difficulties.

**Table 1.** Clinical features associated with intragenic and CRMs of SATB2

	Intragenic mutations in SATB2								*cis*-Regulatory mutations		
Paper (PMID)	10417281	19668335			17377962	23020937	19170718	18371933	This report	This report	21295280
Case	Case 1	Case 1	Case 2	Case 3	Case 1	ER52725	Case 1	Case 49	Case 1	Case 2	Case 1
Mutation	46, XX, t(2;7)(q33;p21) *de novo* disrupting SATB2- transcription unit chr2:200 309 696–200 319 784 hg19	Deletion only involving *SATB2*: chr2:200 128 960–200 312 555 hg19	Deletion only involving SATB2: chr2:200 151 982–200 325 064 hg19	Deletion only involving SATB2: chr2:200 151 782–200 336 956 hg19	Heterozygous *SATB2* c.715C>T; p.R239X; chr2:200 213 881 hg19	Heterozygous SATB2 chr2: g.200 213 455A→C; p.Val381Gly *de novo*	BP disrupting *SATB2-*transcription unit; 46,XY,t(2;14)(q33;q22) *de novo*; BP chr2:200 166 659–200 166 954 hg19	46,XX,t(2;10)(q33;q21.2) *de novo*: BP disrupting SATB2-transcription unit chr2:200 166 166–200 203 438 hg19	t(2;11)(q33.1;p13) *de novo*; BP within PLCL1-SATB2 gene desert between chr2:200 000 820 and 200 010 665 hg19	46XY,t(2;3)(q33.1;q26.33) *de novo;* BP within *PLCL1-SATB2* gene desert between chr2:199 380 000 and 199 390 000 hg19	46,XY,t(1;2)(p34;q33) paternal/cosegregating. BP within PLCL1-SATB2 gene desert between chr2:199 214 552 and 199 261 488 hg19
Phenotypic sex	F	F	M	F	M	F	M	?	F	M	M
Birth weight (g)	2950	3884	50th %ile	?	?	3070/−0.65	3200	?	3720	3515	?
Gestation (weeks)	38	?	?	?	?	34	?	?	40	41	?
Age (years)	11	9.7	21	6	36	2.66	0.1	21	24	33	4
Height (cm)	50–75th %ile	25–50th %ile	10th	75th %ile	25th %ile	−0.81	Post-natal growth failure	?	50–75th %ile	?	?
Weight (kg)	?	50–75th %ile	5–10th	50–75th %ile	<3rd %ile	NR	?	?	10th %ile	?	?
OFC (cm)	50th %ile	75th %ile	>98th	2nd %ile	50th %ile	−1.17	?	?	50–75th %ile	?	?
Small mouth	Yes	?	?	?	?	Yes	Yes	?	Yes	Yes	Yes
Cleft palate	Yes	No	Yes	No	Yes	Bifid uvula	Yes	Yes	Yes	Yes	Yes
Micrognathia	?	Yes	Yes	?	Yes	?	Yes	Yes	Yes	Yes	Yes
Obstruct apnea	?	?	?	?	?	?	Yes	?	No	?	?
Feeding difficulties	?	?	Yes	?	?	?	Yes	?	Yes	Yes	?
Prominent nasal bridge	Yes	No	Yes	?	No	No	Yes	?	Yes	Yes	Yes (from photo)
Long nose	Yes	Yes	?	?	Yes	No	Yes	Yes	Yes	Yes	Yes (from photo)
Long columella	Yes	?	?	?	No	No	?	?	Yes	Yes	Yes (from photo)
Arachnodactyly	Yes	No	?	?	?	NR	?	Yes	Yes	Yes	?
Teeth		Delayed eruption of primary dentition	Overcrowded	Fused central incisors	Marked overbite	NR	N/A		Oligodontia	Small teeth in primary dentition now edentulous	Oligodontia
Sat unaided	24	?	?	Late	?	NR	N/A	?	Late	3 years	?
Walked unaided	?	?	2–3 years	Late	?	NR	N/A	?	24 months	5 years	?
Speech delay	Yes	Yes	Yes	No speech	Yes	NR	N/A	Yes	Yes	No speech	?
Intellectual disability	Moderate	IQ <50	Severe	IQ 32	Yes	Yes	Global developmental delay	Yes	Mild	Severe	?
Neuroimaging	?	MRI & CT normal	?	?	CT	Delayed myelination on brain MRI	Agenesis of the corpus callosum and ventriculomegaly	?	See Report	?	?
Seizures					Tonic clonic	No	Yes		No		
Skeletal disorders					Osteopenia, Scoliosis	NR	Osteopenia	Scoliosis		Osteomalcia diagnosed in childhood	?
Other features							Laryngomalacia and absence of the epiglottis	Pointed chin, very sociable, coarse features, high anterior hairline with cowlick	Bilateral strabismus requiring surgery, excellent long-term memory	Contented child aggressive behavior as adult. Severe gastroesophageal reflux and constipation	Malar hypoplasia. Father has the translocation and a similar phenotype
Other BP in translocation cases	7p21 BP maps between *HDAC9* and *TWISTNB*; chr7:19 222 993–19 247 899 hg19	N/A	N/A	N/A	N/A	N/A	14q22 BP maps to between *TMEM260* and *OTX2*; chr14:56 234 451–56 235 572 hg18	10 BP maps between *EGR2* and *NRBF2*; chr10:64 314 352–64 495 348 hg18	11p13 BP maps between *HIPK3* and *KIAA1549L* chr11:33 461 914–33 544 317 hg19	3q26.33 BP maps >27.7 kb 3′ to *SOX2* between chr3:181 460 016 and 181 526 605 hg19	1p34 BP disrupts *FAF1* transcription unit within the first intron
Reference (PMID)	18374296						19170718	18371933	18374296	This report	21295280

**Figure 2.**
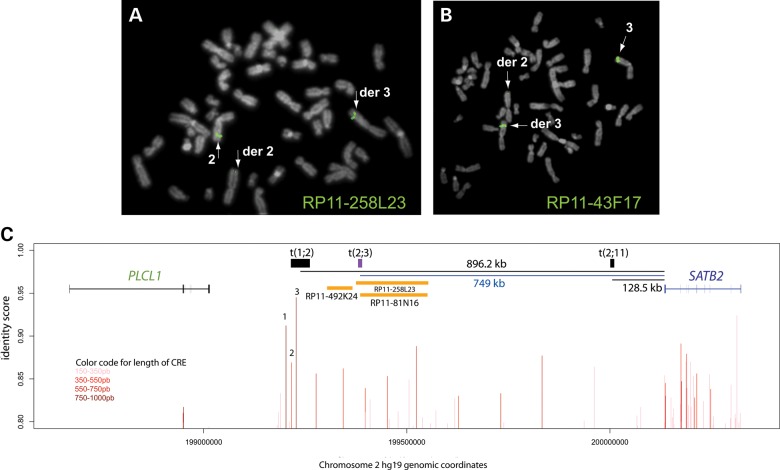
FISH mapping of translocation BPs with potential *cis*-regulatory effect. BP spanning clones in Case 2. (**A**) BAC RP11-258L23 was confirmed as the BP spanning clone on chromosome 2 with signal visible on derivative 2 and derivative 3. (**B**) BAC RP11-43F17 is BP spanning clone on chromosome 3 with signal visible on the derivative 3 and derivative 2. (**C**) Graphical representation of the PLCL1-SATB2 genomic interval using hg19 coordinates. The interval containing the t(2;3) BP is shown as a purple box. Orange bars represent human BAC probes that delineated this BP. BAC RP11-258L23 was found to span the t(2;3) BP which lies ∼749 kb away from SATB2 polyadenylation signal. The intervals containing the BPs for Case 1 (t(2;11)) and Case 3 (t(1;2)) are shown as black boxes. The distance between each of these BP and the SATB2 polyadenylation signal is indicated by the black lines below. The red lines at the bottom of the graph indicate the presence of potential CRE at that genomic coordinate with the color of the line indicating the length of the CRE. The CREs labeled 1, 2 and 3 were chosen for further study as these were potentially disrupted by all of the BPs.

Fluorescence *in situ* hybridization (FISH) mapping of the BPs of this translocation has been reported previously (25). This showed the chromosome 2 BP to lie in the interval of chr2:200 000 820–200 010 665 hg19 (Table 1, Fig. 2) ∼128.5 kb 3′ (centromeric) of the *SATB2* polyadenylation site. The chromosome 11 BP lies in the interval of chr11:33 461 914–33 544 317 hg19 (Table 1) in an intergenic interval between *HIPK3* and *KIAA1549L.* The chromosome 11 intergenic region contained no human-chick conserved potential *cis*-regulatory elements (CREs) using the parameters of >80% identity >150 bp regions using ECR browser (http://ecrbrowser.dcode.org).

#### Case 2 46XY,t(2;3)(q33.1;q26.33) *de novo*

A 33-year-old male with PRS and severe intellectual disability was referred for dysmorphic assessment. He was seen in a secure psychiatric ward, where he was detained for reasons of aggressive behavior. A full examination and accurate growth measurements were not possible because of marked agitation. He had a long, thin face with a prominent nasal ridge, a small mouth, micrognathia (Fig. 1D and E) and bilateral mild arachnodactyly. The clinical similarity between this man and the previously reported *SATB2* translocation cases was noted (see below and Table 1) and a karyotype showed a *de novo* reciprocal translocation, 46XY,t(2;3)(q33.1;q26.33). Metaphase FISH mapping located the 2q33.1 BP to 760 kb 3′ (centromeric) of the *SATB2* polyadenylation site within the gene desert between *SATB2* and *PLCL1* (chr2:199 380 000–199 390 000 hg19) (Supplementary Material, Table S1 and Fig. 2). The 3q26.33 BP mapped to a gene desert 50–80 kb 3′ (telomeric) of *SOX2* (chr3:181 460 016–181 526 605) (Supplementary Material, Table S1) in a region that has not previously been implicated in human disease. *SOX2* heterozygous loss-of-function (het-LOF) mutations are associated with a syndromic form of anophthalmia or severe microphthalmia (26).

#### Re-interpretation of a reported case; 46,XY, t(1;2)(p34;q33)

Ghassibe-Sabbagh *et al.* reported an interesting family in which cleft palate, micrognathia, microstomia and oligodontia segregated with a 46,XY, t(1;2)(p34;q33) in father and son (27). The father's sister had a similar clinical phenotype, but no karyotype information was reported on her. Genome-wide analysis revealed no copy number variation in the affected son. The BP at 1p34 mapped within the first intron of *FAF1*, and investigation of the possible role of this gene in orofacial clefting was the focus of the original report. However, the 2q33 BP-spanning clone (Table 1, Fig. 2) maps within the *PLCL1*-*SATB2* gene desert ∼896 kb 3′ (centromeric) of *SATB2* polyadenylation site (28). This BP location combined with the clinical overlap with Case 1, Case 2 and previously reported cases (see below, Table 1) strongly suggests that these cases have a shared developmental pathogenesis.

### SATB2 het-LOF and CRMs cause the same recognizable clinical syndrome

Eight cases with het-LOF mutations of *SATB2* have been reported (9,11,12,16,17,29). The available clinical features are recorded in Table 1 together with those from the three individuals with potential CRMs. Pre- and post-natal growth appears normal in all cases apart from the low adult weight in the individual with p.R239X. Comparing the assessable features between the het-LOF and CRM cases, intellectual disability is present in 8/8 and 3/3, cleft palate in 6/8 and 3/3, micrognathia in 5/5 and 3/3, long nose in 4/5 and 3/3, small mouth in 3/3 and 3/3 and feeding difficulties in 2/2 and 2/2, respectively. Apparently, heterogeneous dental malformations appear common in both groups. Osteoporosis or osteomalacia was recorded in three of the cases. Only one case (in het-LOF group) had brain malformation, this individual had previously been diagnosed as having Toriello–Carey syndrome (OMIM 217980). It seems likely on the basis of the overlap of clinical features and facial appearance that the individuals with putative-*SATB2* CRM have the same condition as those with *SATB2* het-LOF. This suggests that the translocation BPs result in functional haploinsufficiency in critical-embryonic tissues.

### *PLCL1-SATB2* gene desert contains multiple conserved potential CREs

To identify CREs that may have been disrupted by the translocation BPs, we used a comparative genomics approach to the *PLCL1-SATB2* gene desert (Fig. 2C). Using the parameters of >80% identity >150 bp between the orthologous regions in human and chick genomes, we identified 42 CREs ranging in size from 150 to 933 bp (median 251 bp) and from 80 to 94.5% (median 81.1%) identity. Three of these, CRE1–3, were chosen for analysis as they were disrupted by every BP and based on their large size and high level of cross-species conservation. These elements were named in centromeric to telomeric order; CRE1 (933 bp, 91.2%), CRE2 (473 bp, 86.9%) and CRE3 (909 bp, 94.5%) (for hg19 genomic coordinates, see Supplementary Material, Table S8). Using chromatin immunoprecipitation (ChIP) in combination with quantitative real-time polymerase chain reaction (qPCR), we showed that each of these elements bound p300 and H3K4 monomethylation (H3K4me1) in *Satb2*-expressing cells derived from mouse embryonic pharyngeal arch (MEPA) consistent with enhancer function (30) (Fig. 4A). The Sox9-promoter region was used as a negative control (data not shown) in these experiments as previously reported (7). Given the high concordance of cleft palate and micrognathia in the affected individuals, we hypothesized that *SATB2* may be a target of the transcription factor SOX9 as CRMs affecting *SOX9* are a significant cause of PRS (7). A motif-based search of the elements revealed four conserved sites that would-be predicted to bind SOX9, two in CRE1 and one each in CRE2 and CRE3 (Supplementary Material, Fig. S1). The ChIP–qPCR analysis of Sox9 binding in MEPA cells was consistent with the prediction with all three elements showing binding to Sox9 with CRE1 showing significantly greater fold enrichment over the IgG controls compared with CRE2 and CRE3 (Fig. 4B). A sequence located at the 3′ end of *Col2a1* that had been previously used as a negative control for Sox9 ChIP (31) likewise showed no enrichment compared with IgG in Sox9 ChIP from MEPA cells (data not shown).

### *Plcl1-Satb2* transcription-dependent chromatin de-condensation

**Figure 3.**
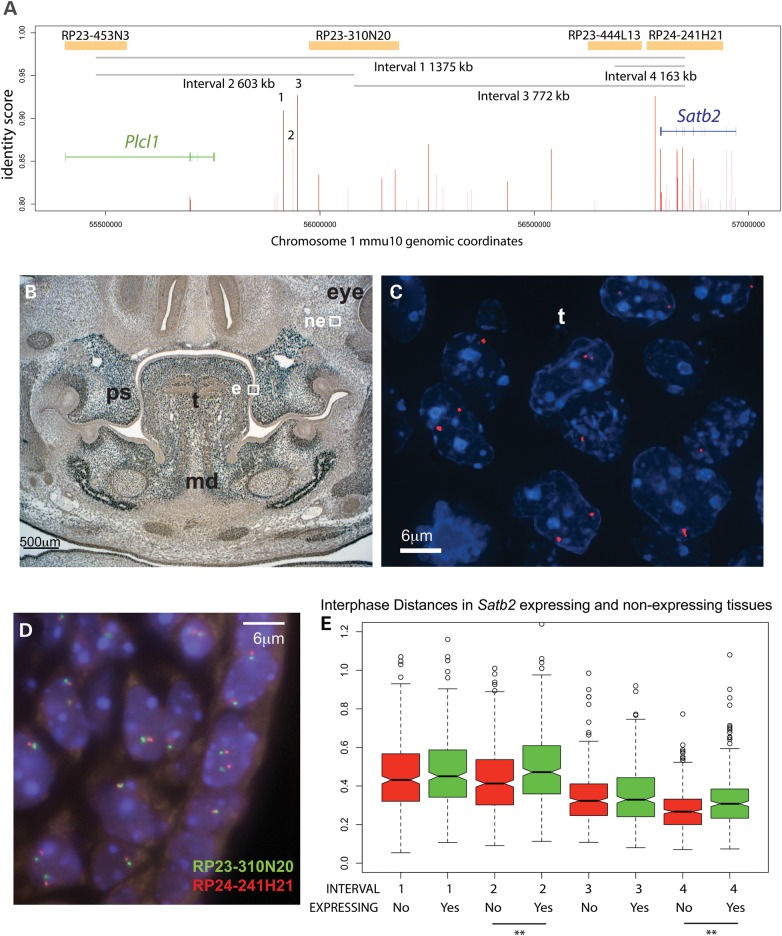
Interphase FISH measurement of *Plcl1-Satb2*interval in expressing and non-expressing tissues. 3D distance measurements between interphase FISH signals in sections of mouse embryonic tissues reveals localized tissue-specific chromatin decompaction at the Satb2 locus. (**A**) Genomic organization of the Satb2 locus in mouse (mm9). Orange bars represent mouse BAC probes used in 3D distance measurements. Gray lines represent the distance of each interval, 1–4, between BAC probes. Red lines represent potential CREs. In particular, CREs 1, 2 and 3 are potentially disrupted by each reported BP. (**B**) Confirming Satb2 expression: immunohistochemistry in the branchial arch region of a wild-type mouse embryo at 13.5 dpc (coronal section, 4 μm thick). Staining is observed in the palatal shelves (ps), tongue (t) and mandible (md). Expressing (E) and non-expressing (NE) areas were examined at 100× magnification in adjacent sections, the former corresponding to region represented by the RNA FISH image in (**C**). (C) RNA FISH signals (in red) show Satb2 expression at the cellular level in the left palatal shelf and tongue. (**D**) DNA FISH signals of mouse BAC probe pair RP23-310N0 and RP24-241H21 taken from the right palatal shelf. (**E**) Optical sectioning of individual nuclei in sections of 13.5 gestational days mouse embryonic tissue in Satb2-expressing and non-expressing areas allowed distances between mouse BAC probe signals, four probe pairs in total, to be measured in 3D. The black lines above a double asterisk indicate a significant transcription-dependent chromatin decondenzation based on a 95% confidence interval (CI) (see Supplementary Material, Tables S2–S4).

Although CRE1–3 have characteristics of enhancers involved in *cis*-regulation of developmental genes, it was necessary to establish which gene or genes these enhancers were targeting. As a first step, we examined the degree of chromatin condensation *in vivo* within the *Plcl1-Satb2* gene desert to determine if the chromatin state changed in *Satb2*-expressing cells compared with those that are transcriptionally silent. We measured the 3D distance between pairs of bacterial artificial chromosome (BAC) probes *in vivo* using DNA FISH (Fig. 3D) on developing craniofacial tissues in 13.5 days post coitum (dpc) mouse embryos. BACs containing *Plcl1* (RP23-453N3), the region orthologous to the t(2;3) (RP23-310N20) and t(2;11) (RP23-444L13) and *Satb2* (RP24-241H21) were used for the analysis (Fig. 3A and D). Immunohistochemistry was used to identify Satb2-expressing and non-expressing tissue (Fig. 3B), and RNA FISH was used to confirm that both alleles of *Satb2* were active in each cell in the expressing tissues (Fig. 3C). Satb2 is expressed in the palatal shelves, tongue and mandible (Fig. 3B and C).

The 3D interphase distance across the gene desert (*Plcl1* to *Satb2*; Interval 1) was similar in all tissues examined as was that between t(2;3) BP and *Satb2* (Interval 3) (Fig. 3E, Supplementary Material, Tables S2–S4). However, the mean distance between *Plcl1* and the t(2;3) BP (Interval 2) was significantly larger in Satb2-expressing tissue (Fig. 3E and Supplementary Material, Tables S2–S4) as was that between the t(2;11) region and *Satb2* (Interval 4). These results strongly support *Satb2* as the target of the enhancer activity associated with this locus.

### CRE2 drives Sox9-dependent *Satb2*-like expression in transgenic zebrafish

**Figure 4.**
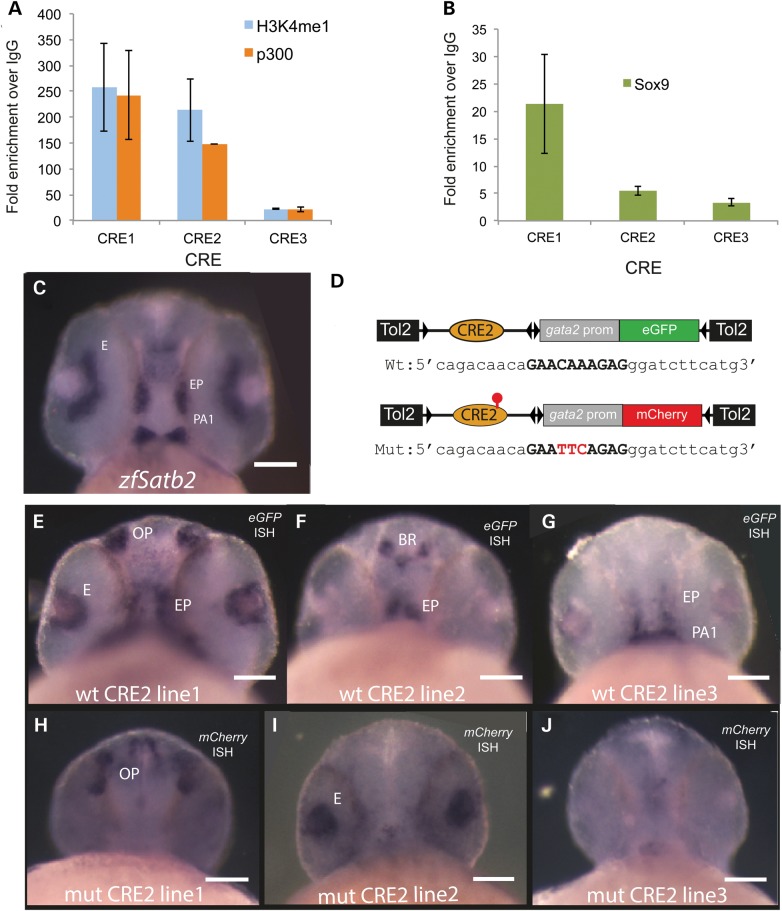
Expression patterns driven by wild-type and mutant SATB2 CRE2 in zebrafish reporter transgenics. (**A**) Graph of the fold enrichment over IgG using ChIP for H3K4 monomethylation (H3K4me1) and p300 of CRE1–3 in MEPA cells showing that each of the elements binds to p300 and H3K4me1, marks of enhancer function significantly above background. (**B**) Graph of the fold enrichment over IgG using Sox9 antibody measures using qPCR for CRE1–3 in MEPA cells showing that each of the elements also binds SOX9 above background level. (**C**) mRNA *in situ* hybridization using an antisense probe for *zfSatb2* showing expression in the developing pharyngeal arch (PA1), ethmoid plate (EP) and eye (E). (**D**) Cartoon of the constructs used to make the stable transgenic lines. (**E–G**) mRNA *in situ* hybridization using an antisense probe for *eGFP* showing reporter gene expression driven by CRE2 in embryos collected from stable zebrafish reporter transgenic line. CRE2 drives eGFP expression in the developing eye (E), ethmoid plate (EP), olfactory placode (OP), brain (BR), and pharyngeal arch (PA1). The same element bearing mutations in the predicted SOX9-binding sites (**H**–**J**) fails to drive a similar expression pattern. The reporter activity in the mutant lines was detected using an antisense probe for *mCherry*.

To determine if CRE1–3 did indeed have *SATB2* enhancer function *in vivo*, we employed reporter transgenic analysis in zebrafish. The reporter constructs consisted of the full-length human CRE upstream of the mouse *Gata2* minimal promotor driving eGFP. At least three stable lines were established from each CRE, and embryos were screened at 24 h and the tissue distribution and consistency of reporter activity recorded by fluorescence microscopy. Only CRE2 resulted in transgenic lines that each gave an expression pattern which accurately represented a subset of endogenous *zfSatb2* site- and stage-specific expression. However, CRE1 drove expression in the olfactory placode at 48 h, which is a site of *Satb2* expression, although endogenous *Satb2* expression is not detectable until 96 h (Supplementary Material, Fig. S2). The CRE2 reporter activity is represented in Figure 4E–G as whole-mount *in situ* hybridization (WISH) using an antisense riboprobe to *eGFP* transcript. Each line shows expression in the ethmoid plate (Fig. 4E–G). In CRE2-line3, there is also expression in the first pharyngeal arch (Fig. 4E) and, in CRE2-line1, there is expression in the retina that is similar to endogenous *zfSatb2*.

To determine the role that SOX9 plays in driving the craniofacial expression in the CRE2 element, we introduced a mutation that was predicted to ablate the single conserved *SOX9-*binding site in this element. The construct was otherwise identical apart from the fact that mCherry replaced eGFP as the fluorescent reporter. WISH using an antisense riboprobe to *mCherry* transcript showed that none of the stable lines made using this construct resulted in an expression pattern that encompassed a subset of endogenous *zfSatb2* (Fig. 4H–J, Supplementary Material, Table S9). This observation is consistent with the notion that SOX9 is driving the craniofacial expression of Satb2 via interaction with the CRE2 enhancer.

## DISCUSSION

Here, we show that translocation BPs that lie in the gene desert 3′ of SATB2 have a clinical consequence which mimics het-LOF mutations of *SATB2.* This suggests that the translocation BPs produce functional haploinsufficiency in, at least, a subset of developmental tissues with dosage sensitivity for this gene product. The best-studied example of the *cis*-regulatory ‘switch off’ phenomenon has been associated with aniridia (MIM 106210), a panocular developmental eye disorder in which most cases are due to het-LOF mutations in *PAX6*. A proportion of cases that are clinically indistinguishable from those with nonsense mutations result from deletions or translocation BPs located 3′ to the gene (32). A similar phenomenon has also been observed in holoprosencephaly (MIM 142945), which can be caused by het-LOF mutations in *SHH* or by deletions or translocation BPs in a gene desert 5′ to the gene (33). The mechanism by which the gene is silenced in these position effects is not yet clear. The most plausible explanation is that these chromosomal aberrations disrupt the *cis*-regulatory landscape of the target gene via loss of enhancers or insulator elements.

Comparative genomics has provided a powerful tool for identifying non-coding regions of the genome that are under purifying selection with many of these elements thought to be involved in *cis*-regulation of developmental genes (34,35). The identification of specific chromatin-associated proteins as marks of enhancer activity has been another effective approach in the identification of sequences that are critical for site- and stage-specific developmental expression of individual genes (36). Here, both approaches were used in combination with the human genetic data to identify three CREs that are located 900 kb or more 3′ of *SATB2.* Like *SATB2* itself, these CREs show remarkable levels of nucleotide identity across evolution. Each has chromatin marks consistent with enhancer function. CRE2 and CRE1 indeed show consistent site- and stage-specific developmental activity in reporter transgenic assays in zebrafish that at least partly overlaps with the expression pattern of *zfSatb2*. However, recent work has shown that not all enhancer sequences show evolutionary conservation so it is unlikely that we have identified all significant CREs (37).

While efficient methods now exist to identify DNA regions with the characteristics of CREs robust methods for identifying the target of the regulation have yet to be validated. Here, we have taken the phenotypic similarity of the translocation cases with the het-LOF mutations as an indication that *SATB2* may be the target of CRE1–3. We have two further pieces of evidence that CRE2, at least, is an *SATB2* enhancer. The region containing these distal enhancers shows *SATB2* transcription-dependent chromatin de-condensation, a phenomenon that is common to many developmentally-regulated loci (7,38). We have also shown that CRE2 drives expression in multiple independent stable transgenic lines that drive a subset of the endogenous *zfSatb2* activation domains.

Given the combination of cleft palate and micrognathia in *SATB2* haploinsufficiency cases, there is a clear overlap with the spectrum of craniofacial malformations seen in genetically characterized forms of PRS. We have recently shown that a significant proportion of isolated autosomal dominant PRS is the consequence of CRMs affecting the developmental expression of *SOX9* (7,39). Some affected individuals within the SOX9 CRM families had micrognathia as their sole phenotype. We therefore hypothesized that some or all of the *cis*-regulation of *SATB2* critical for craniofacial development may be driven by binding of *SOX9. In silico* analysis suggested that there were SOX9-binding sites in each of the elements we had chosen to study and ChIP-analysis using a SOX9 antibody suggested that there is binding in cells derived from the mouse embryonic maxilla and mandible. The fact that disruption of the predicted SOX9-binding element within CRE2 resulted in a loss of reporter activity supported our original hypothesis.

The results presented here are consistent with the notion that the translocation BPs have disrupted the long-range *cis*-regulation of *SATB2* by SOX9. Identification of small deletions or point mutations within these elements in cases with PRS would certainly strengthen the case for a critical role for CRE2. There are other possible explanations for the association we have described; the translocations have removed an insulator region which has resulted in ‘heterochromatinization’ of the locus. One or more of these elements could function both as an enhancer and an insulator.

This work has confirmed the existence of a clinically recognizable *SATB2*-associated syndrome and has provided evidence that the target region for deleterious mutations affecting this gene may be much larger than previously thought.

## MATERIALS AND METHODS

### Case ascertainment

Case 1 and Case 2 were ascertained via routine clinical genetics investigations in a regional genetics service laboratory using conventional cytogenetic analysis of metaphase chromosomes from peripheral blood cells. The FISH mapping experiments were performed under ethical approval provided by the UK Multiregional Ethics Committee (Reference: 06/MRE00/77) and imaging and psychometry under ethical approval references London–Queen Square 01/N078 and 01/N120.

### 2D metaphase FISH and BP mapping

Metaphase FISH analysis was performed on patient-derived lymphoblastoid cell line using BAC and P1-derived artificial chromosome (PAC) clones from the Wellcome Trust Sanger Institute (Cambridge, UK) or BACPAC resources (Oakland, CA, USA) (http://www.chorio.org/bacpac/). Probes labeled with biotin-16-dUTP or digoxigenin-11-dUTP (Roche, Indianapolis, IN, USA) were prepared, hybridized and detected as described previously (40). For each hybridization, five metaphases or more were analyzed using a Zeiss Axioplan 2 fluorescence microscope equipped with a triple band-pass filter (#83000 for DAPI, FITC and Texas Red; Chroma Technology, Brattleboro, VT, USA). Images were collected using a cooled CCD camera (Photometrics, CoolSNAP HQ) and analyzed using SmartCapture software (Digital Scientific). Long-range PCR was used to generate probes for fine BP mapping. Primers were designed using Primer 3 (http://frodo.wi.mit.edu/cgi-bin/primer3/primer3_www.cgi).

### 3D interphase RNA and DNA FISH on embryonic tissue sections

Wild-type mouse embryos at 13.5 dpc were fixed, paraffin-embedded and sectioned at 4 µm. Adjacent slides were used for RNA FISH, DNA FISH and immunohistochemistry. DNA FISH was performed as described (41). Mouse BAC probes were obtained from BACPAC resources Children's Hospital Oakland Research Institute. BAC clones were labeled with digoxigenin-11-dUTP (dig) or the direct fluorescent label Orange-dUTP (Enzo Life Sciences). Anti-dig antibody conjugated to fluorescein (Roche) was used to visualize the dig-labeled probes. RNA FISH was performed as reported (42) using a pool of six different 2.5 kb intronic regions of *Satb2* amplified from BAC RP24-241H21 labeled with Orange-dUTP (Enzo Life Sciences). Two hundred and fifty nanograms of labeled probe were added to the tissue sections in a final volume of 10 µl hybridization buffer. After overnight incubation at 37°C, slides were washed; 3 min in 2× SSC/50% formamide at RT, 3 min in 2× SSC/50% formamide at 37°C, 3 min in 2× SSC at RT and 3 min in 4× SSC/0 1% Tween. Slides were mounted in DAPI/Vectashield (Vector Laboratories, Burlingame, CA, USA). The imaging system comprises a Hamamatsu Orca AG CCD camera (Hamamatsu Photonics (UK) Ltd, Welwyn Garden City, UK), Zeiss Axioskop fluorescence microscope with plan-neofluar or plan apochromat objectives, a Lumen 200W metal halide light source (Prior Scientific Instruments, Cambridge, UK) and Chroma #83000 triple band-pass filter set (Chroma Technology Corp., Rockingham, VT, USA) with the excitation filters installed in a prior motorized filter wheel. A piezoelectrically driven objective mount (PIFOC model *P*-721, Physik Instrumente GmbH & Co, Karlsruhe, Germany) was used to control movement in the z-dimension. Images were deconvolved using the constrained iterative algorithm of Volocity 6 (Perkinelmer Inc, Waltham, MA, USA) and inter-spot distances were calculated using the Velocity 6 quantitation module.

### SATB2 antibody production and validation

To express the C-terminal portion of the human SATB2, we amplified the corresponding fragment from a full-length *SATB2* cDNA clone. An N-terminal HIS-tag was added using Gateway Technology and the fusion protein expressed from BL21 (DE3)PLysS competent cells. The protein was purified using Ni-NTA purification system (Invitrogen) and used for polyclonal antibody production (CovalAb UK). The resulting rabbit serum was affinity purified using standard methods. The antibody was tested by western blot on MEPA cells transfected with full-length human *SATB2* cDNA with an N-terminal GFP tag in vector pcDNA-DEST 53 (Invitrogen) using the MP-100 Microporator (Digital Bio). Successful transfection of SATB2-GFP was confirmed by western blot using a 1:2000 dilution of affinity purified Satb2 polyclonal antibody and a 1:1000 dilution of monoclonal anti-GFP antibody. Immunohistochemistry was also performed on 13.5 dpc wild-type and *Satb2*-null mouse embryo sections (19).

### Immunohistochemistry

Immunohistochemistry was performed on 4 µm paraffin sections using the VECTASTAIN Elite ABC Kit (Rabbit IgG) protocol (Vector Laboratories) with some adaptation. For antigen unmasking, slides were boiled in 10 mm tri-sodium citrate (pH 6) and subsequently blocked in 1% horse serum/1% goat serum (PBS Tween). Sections were incubated in a 1:100 dilution of polyclonal antibody against Satb2 for 30 min. Secondary antibody incubation and further steps were carried out according to protocol. Sections were detected using ImmPACT DAB (Vector Laboratories).

### Identification of CREs and ChIP

Conserved non-coding elements in the PLCL1-SATB2 gene desert and the orthologous region in the mouse genome were identified on the basis of conservation in the chick genome of >80% >150 bp using the ECR browser (http://ecrbrowser.dcode.org). ChIP was performed as described (43), with several modifications (details available on request). Chromatin samples from MEPA cells were immunoprecipitated with 5 µg of H3K4me1 (ab8895, Abcam), 10 µg of p300(C-20:sc585, Santacruz), 10 µg of SOX9 (AB5809, Milipore) or 10 µg of IgG (ab46540, Abcam) antibodies, and the immune complexes were collected by incubating with protein-A-Dynabeads. The beads were washed and the immune complexes were eluted with 50 mm Tris, pH 8.0, 1 mm EDTA and 1% SDS at 65°C for 10 min, adjusted to 200 mm NaCl and incubated at 65°C overnight to reverse the cross links. After successive treatments with 10 µg/ml RNase A and 20 µg/ml proteinase-K, the samples were eluted into 50 µl H_2_O using the QIAquick Spin Gel Purification Kit (Qiagen). The ChIP–qPCR experiments were carried out using SYBR Green PCR Master Mix and LightCycler 480 (Roche) platform according to manufacturer's instructions using input and ChIP DNA as a template. The primers used for the qPCR are shown in Supplementary Material, Table S6.

### Whole-mount *in situ* hybridization

The zebrafish *zfSatb2* antisense probe was made using T7 polymerase *in vitro* transcription as previously described (44). mCherry and eGFP probes were made with T7 using a template that was PCR-amplified using the primers listed in Supplementary Material, Table S7 from the reporter transgenic constructs (see below). mRNA *in situ* hybridization was performed as previously described (45). Embryos selected for imaging were mounted in 1% low-melting agarose. Images were taken on Nikon macroscope AZ100 and processed using iVision software.

### Generation of zebrafish transgenic lines

The cloning of the CRE1–3 and the generation of the zebrafish transgenic lines bearing the enhancer-reporter constructs was carried out as described (46). Embryos (F1) from the founders showing the best representative expression pattern for each construct were selected and processed for WISH, as described above. At least four-independent transgenic lines were analyzed for each element, and the images shown are representative of expression patterns observed for at least 10 embryos from each independent transgenic line. The details of the primers used for amplification of the CREs are listed in Supplementary Material, Table S7.

## SUPPLEMENTARY MATERIAL

Supplementary Material is available at *HMG* online.

## FUNDING

This work was supported through the UK Medical Research Council (MRC) core program funding within the MRC Human Genetic Unit. We are grateful to the Wolfson Trust and the Epilepsy Society for supporting the Epilepsy Society MRI scanner. This work was, in part, undertaken at UCLH/UCL who received a proportion of funding from the Department of Health's NIHR UCLH Biomedical Research Centre funding scheme. This research was also supported by a joint grant from the NIHR Biomedical Research Centres at UCLH/UCL, Great Ormond Street Hospital for Children/UCL Institute of Child Health and Moorfields Eye Hospital/UCL Institute of Ophthamology (Grant No.147) and the MRC. Funding to pay the Open Access publication charges for this article was provided by the University of Edinburgh Institutional Publication Fund (IPF).

## Supplementary Material

Supplementary Data
