# Supplementary material for: Disruption of SATB2 or its long-range cis-regulation by SOX9 causes a syndromic form of Pierre Robin sequence
Source: Hum Mol Genet. 2013 Dec 20;23(10):2569–79. doi: 10.1093/hmg/ddt647 (PMC3990159; doi:10.1093/hmg/ddt647)
Supplement: Supplementary Data [file supp_23_10_2569__index.html]

Disruption of SATB2 or its long-range cis-regulation by SOX9 causes a syndromic form of Pierre Robin sequence — Disruption of SATB2 or its long-range cis-regulation by SOX9 causes a syndromic form of Pierre Robin sequence — Supplementary Data 

# Disruption of *SATB2* or its long-range *cis*-regulation by SOX9 causes a syndromic form of Pierre Robin sequence

## Supplementary Data

Supplementary Data

**Files in this Data Supplement:**

- Supplementary Figure 1 - pdf file
- Supplementary Figure 2 - pdf file
- Supplementary Data - Docx file
