# Supplementary figures and images for: Disruption of SATB2 or its long-range cis-regulation by SOX9 causes a syndromic form of Pierre Robin sequence
Source: Hum Mol Genet. 2013 Dec 20;23(10):2569–79. doi: 10.1093/hmg/ddt647 (PMC3990159; doi:10.1093/hmg/ddt647)

*a.* **wrarAACAAATgrw:**

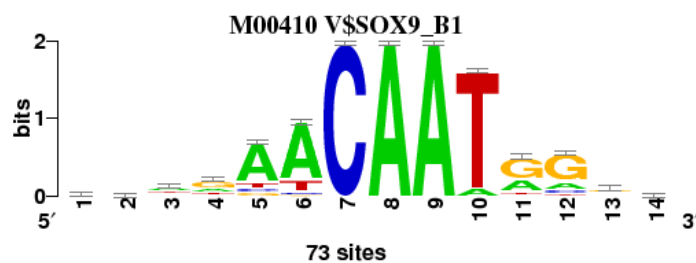

*b.* **rwwCARAGgvm:**

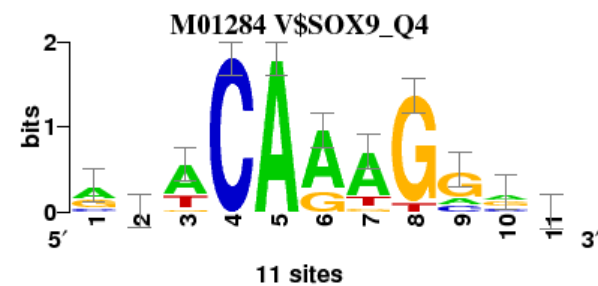

*c.*

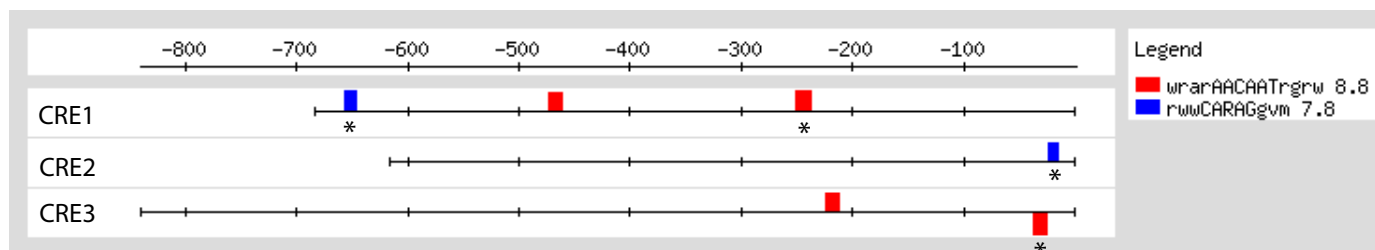

*d.*

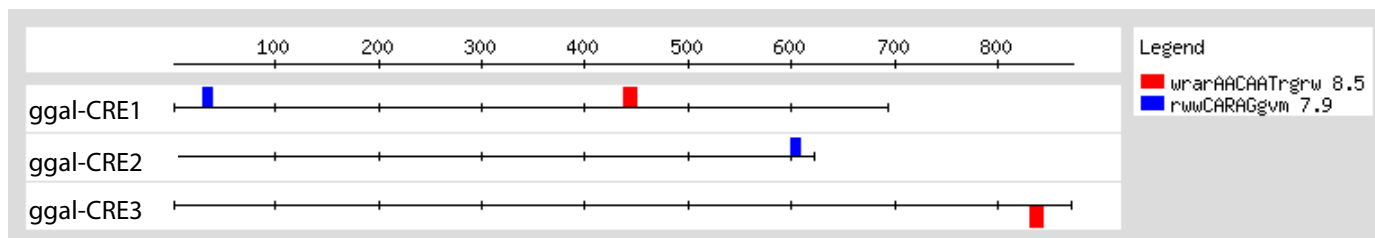

Supplement: Supplementary Data [file supp_ddt647_ddt647supp_fig1.pdf]

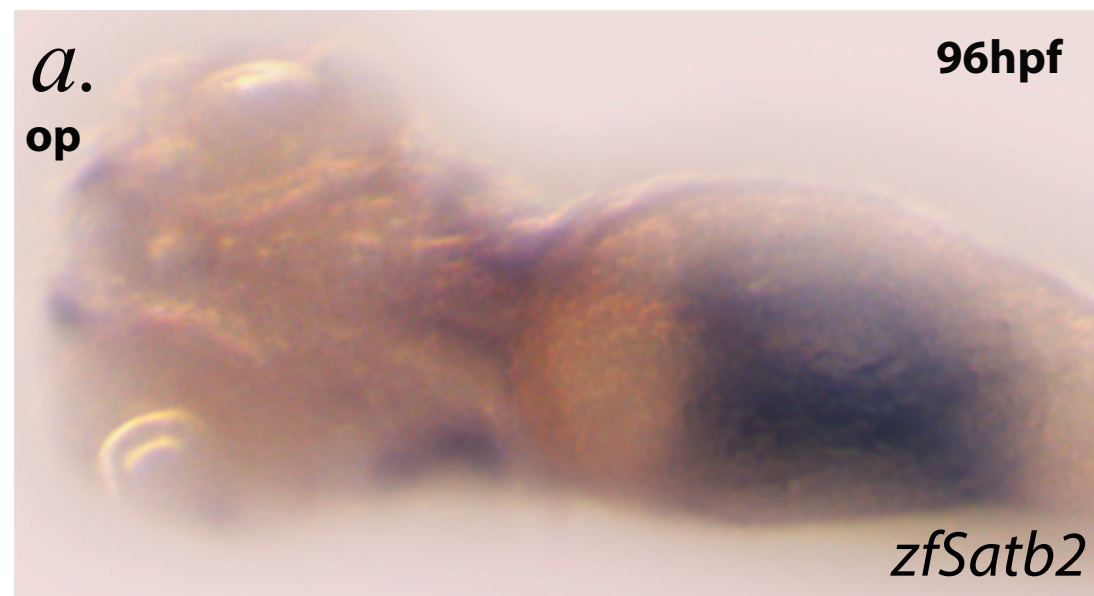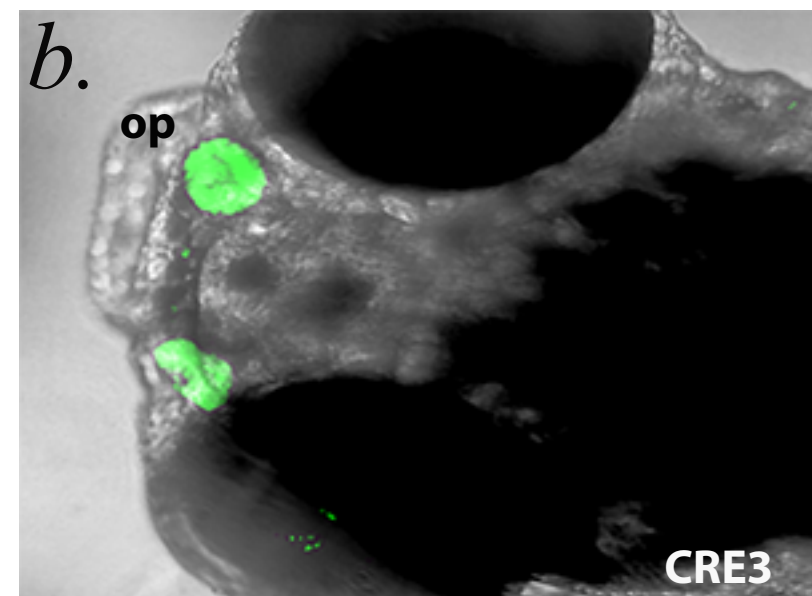

Supplement: Supplementary Data [file supp_ddt647_ddt647supp_fig2.pdf]
