# Supplementary material for: Disruption of SATB2 or its long-range cis-regulation by SOX9 causes a syndromic form of Pierre Robin sequence
Source: Hum Mol Genet. 2013 Dec 20;23(10):2569–79. doi: 10.1093/hmg/ddt647 (PMC3990159; doi:10.1093/hmg/ddt647)
Supplement: Supplementary Data [file supp_ddt647_ddt647supp.docx]

# SUPPLEMENTAL DATA

# Disruption of *SATB2* or its long-range *cis*-regulation by SOX9 causes a syndromic form of Pierre Robin Sequence

Jacqueline K Rainger^1^, Shipra Bhatia^1^, Hemant Bengani^1^, Philippe Gautier^1^, Joe Rainger^1^, Matt Pearson^1^, Morad Ansari^1^, Jayne Crow^2^, Felicity Mehendale^3^, Bozena Palinkasova^4^, Michael J Dixon^4^, Pamela J Thompson^5,6^, Mar Matarin^5^, Sanjay M Sisodiya^5,6^, Dirk A Kleinjan^1^, David R FitzPatrick^1*^

*Supplemental Figure Legends*

**Figure S1 *In silico* analysis of SOX9 binding sites** Transcription factor binding site analysis of CRE1-3 using two different Sox9 matrices from Transfac. A. Graphical representation of matrix M00410 (V$SOX9_B1) which is derived from 73 SELEX-derived binding sites collection. B. Graphical representation of matrix M01284 (V$SOX9_Q4) is a collection of 11 binding sites from published sources that have been validated using gel shift/gel supershift assays. C,D Cartoon representation of the position of the positive search results using both matrices (coloured red and blue respectively) in human CRE1-3 and the orthologous regions in chicken (ggal_CRE1, ggal_CRE2, ggal_CRE3). The black lines represent the size of the elements. The SOX9 binding sites in sense orientation are shown above the line and those that are antisense below. The binding sites that are conserved with the chick element are indicated with an asterix in C, 2 of 6 sites in humans are not conserved in chicken.

**Figure S2 zfSatb2 expression and reporter transgenic activity in the olfactory placode. A** Whole mount in situ hybridization using *zfSatb2* probe in 96 hours post fertilization embryo showing expression (blue stain) in the olfactory placode (op). B. GFP reporter transgenic activity in the olfactory placode from representative 48hr embryo from stable transgenic lines made using human CRE3.

| **Table S1: Most informative BAC probes in FISH mapping of Case 2** | | | | | | | | | | | | | | | | | | | |  |  |
| --- | --- | --- | --- | --- | --- | --- | --- | --- | --- | --- | --- | --- | --- | --- | --- | --- | --- | --- | --- | --- | --- |
| **Clone name** | | | **Band** | | **Location (Hg19)** | | | | | **2** | | **der(2)** | | **der(3)** | | **Position relative to breakpoint** | | | |  |  |
| RP11-492K24 | | | 2q33.1 | | 199,303,754-199,366,427 | | | | | + | | + | | - | | Proximal | | | |  |  |
| RP11-258L23 | | | 2q33.1 | | 199,374,989-199,553,049 | | | | | + | | + | | + | | BPS | | | |  |  |
| RP11-81N16 | | | 2q33.1 | | 199,385,252-199,551,028 | | | | | + | | - | | + | | Distal | | | |  |  |
|  | | |  | |  | | | | | **3** | | **der(3)** | | **der(2)** | |  | | | |  |  |
| RP11-335I3 | | | 3q26.33 | | 181,118,937-181,291,391 | | | | | + | | + | | - | | Proximal | | | |  |  |
| RP11-43F17 | | | 3q26.33 | | 181,367,524-181,526,609 | | | | | + | | + | | + | | BPS^a^ | | | |  |  |
| RP11-416O18 | | | 3q26.33 | | 181,694,258-181,870,575 | | | | | + | | - | | + | | Distal | | | |  |  |
| **Clone name** | | | **Band** | | **Location (Hg19)** | | | | | **2** | | **der(2)** | | **der(10)** | | **Position relative to breakpoint** | | | |  |  |
| RP11-505H14 | | | 2q33.1 | | 199,927,660-200,096,298 | | | | | + | | + | | - | | Proximal | | | |  |  |
| RP11-486F17 | | | 2q33.1 | | 200,097,299-200,281,516 | | | | | + | | + | | + | | BPS | | | |  |  |
| WI2-1367G19 | | | 2q33.1 | | 200,166,166-200,203,438 | | | | | + | | + | | + | | BPS | | | |  |  |
| RP11-530J6 | | | 2q33.1 | | 200,281,517-200,411,579 | | | | | + | | - | | + | | Distal | | | |  |  |
|  | | |  | |  | | | | | **10** | | **der(10)** | | **der(2)** | |  | | | |  |  |
| RP11-436D10 | | | 10q21.3 | | 64,550,274-64,625,545 | | | | | + | | + | | - | | Proximal | | | |  |  |
| RP11-132E18 | | | 10q21.3 | | 64,740,494-64,753,753 | | | | | + | | + | | + | | BPS | | | |  |  |
| RP11-144G16 | | | 10q21.3 | | 64,753,754-64,929,336 | | | | | + | | - | | + | | Distal | | | |  |  |
|  | | |  | |  | | | | |  | |  | |  | |  | | | |  |  |
| **Table S2. Pooled results from two 13.5 d.p.c. mouse embryos, A and B.** | | | | | | | | | | | | | | | | | | | | | |
| **BAC** | | | | **453N3-241H21** | | | | **453N3-310N20** | | | | | **310N20-241H21** | | | | | **444L13-241H21** | | | |
| **Region** | | | | ***Plcl1 - Satb2*** | | | | ***Plcl1 - t(2;3)*** | | | | | **t(2;3) - *Satb2*** | | | | | **t(2;11) - *Satb2*** | | | |
| **Genomic distance (kb)** | | | | **1.4 Mb** | | | | **595 kb** | | | | | **788 kb** | | | | | **194 kb** | | | |
| **Stage** | **Tissue** | **Satb2 expression** | | **Nuclei** | | **Mean 3D separation (nm)** | **95% CI; ±** | **Nuclei** | **Mean 3D separation (nm)** | | **95% CI; ±** | | **Nuclei** | | **Mean 3D separation (nm)** | | **95% CI; ±** | **Nuclei** | **Mean 3D separation (nm)** | | **95% CI; ±** |
|  |  |  |  |  |  |  |  |  |  |  |  |  |  |  |  |  |  |  |  |  |  |
|  |  |  |  |  |  |  |  |  |  |  |  |  |  |  |  |  |  |  |  |  |  |
| 13.5 d.p.c. | Palatal shelf/Maxillary mesenchyme | + | | 345 | | 473 | 20 | 414 | 496 | | 18 | | 333 | | 354 | | 16 | 282 | 326 | | 16 |
| 13.5 d.p.c. | Periocular mesenchyme | - | | 382 | | 458 | 18 | 417 | 437 | | 17 | | 348 | | 342 | | 16 | 385 | 279 | | 11 |

| **Table S3. Mouse A.** | | | | | | | | | | | | | | |
| --- | --- | --- | --- | --- | --- | --- | --- | --- | --- | --- | --- | --- | --- | --- |
| **BAC** | | | **453N3-241H21** | | | **453N3-310N20** | | | **310N20-241H21** | | | **444L13-241H21** | | |
| **Region** | | | ***Plcl1 - Satb2*** | | | ***Plcl1 - t(2;3)*** | | | **t(2;3) - *Satb2*** | | | **t(2;11) - *Satb2*** | | |
| **Genomic distance (kb)** | | | **1.4 Mb** | | | **595 kb** | | | **788 kb** | | | **194 kb** | | |
| **Stage** | **Tissue** | **Satb2 expression** | **Nuclei** | **Mean 3D separation (nm)** | **95% CI; ±** | **Nuclei** | **Mean 3D separation (nm)** | **95% CI; ±** | **Nuclei** | **Mean 3D separation (nm)** | **95% CI; ±** | **Nuclei** | **Mean 3D separation (nm)** | **95% CI; ±** |
|  |  |  |  |  |  |  |  |  |  |  |  |  |  |  |
|  |  |  |  |  |  |  |  |  |  |  |  |  |  |  |
| 13.5 d.p.c. | Palatal shelf/Maxillary mesenchyme | + | 120 | 448 | 29 | 202 | 509 | 26 | 166 | 332 | 19 | 175 | 308 | 19 |
| 13.5 d.p.c. | Periocular mesenchyme | - | 155 | 433 | 26 | 219 | 452 | 25 | 137 | 337 | 29 | 165 | 260 | 14 |

| **Table S4. Mouse B.** | | | | | | | | | | | | | | |
| --- | --- | --- | --- | --- | --- | --- | --- | --- | --- | --- | --- | --- | --- | --- |
| **BAC** | | | **453N3-241H21** | | | **453N3-310N20** | | | **310N20-241H21** | | | **444L13-241H21** | | |
| **Region** | | | ***Plcl1 - Satb2*** | | | ***Plcl1 - t(2;3)*** | | | **t(2;3) - *Satb2*** | | | **t(2;11) - *Satb2*** | | |
| **Genomic distance (kb)** | | | **1.4 Mb** | | | **595 kb** | | | **788 kb** | | | **194 kb** | | |
| **Stage** | **Tissue** | **Satb2 expression** | **Nuclei** | **Mean 3D separation (nm)** | **95% CI; ±** | **Nuclei** | **Mean 3D separation (nm)** | **95% CI; ±** | **Nuclei** | **Mean 3D separation (nm)** | **95% CI; ±** | **Nuclei** | **Mean 3D separation (nm)** | **95% CI; ±** |
|  |  |  |  |  |  |  |  |  |  |  |  |  |  |  |
|  |  |  |  |  |  |  |  |  |  |  |  |  |  |  |
| 13.5 d.p.c. | Palatal shelf/Maxillary mesenchyme | + | 225 | 487 | 26 | 212 | 484 | 23 | 167 | 375 | 25 | 107 | 356 | 29 |
| 13.5 d.p.c. | Periocular mesenchyme | - | 227 | 473 | 25 | 198 | 421 | 24 | 211 | 345 | 19 | 220 | 293 | 15 |

| **Table S5: Primers Used to Generate RNA FISH Probe** | | | |
| --- | --- | --- | --- |
| **Primer name (position in *Satb2* gene)** | **Forward 5’ to 3’** | **Reverse 5’ to 3’** | **BAC used for PCR** |
| Intron_2_Satb2_mm | GGAAGTCAAATCCAGGTTGC | TGTGTCCACAGTCCATGTCC | RP24-186A3 |
| Intron_4_Satb2_mm | TCCAAGTGGTTGCCTTTCTT | GCTACACTGGGAAGCTCTGC | RP24-241H21 |
| Intron_5_Satb2_mm | AGGCTTCTGGCAAGTTTGAA | AATTTGCCATCTGATCTTTCTC | RP24-241H21 |
| Intron_6_Satb2_mm | TGCCTAGGAGACACTTGCTG | GCATTGCACCACTCTTCCTT | RP24-241H21 |
| Intron_7_Satb2_mm | GAGGTAACCCTGTGCTGTGG | CAGCAGTGGTGGAATTTGTG | RP24-241H21 |
| Intron_8_Satb2_mm | CCATCGGAGTTCAGAGGAGA | CCATTTCACCGACAACCTCT | RP24-241H21 |

| **Table S6: Primers used for the qPCR of CREs** | | |
| --- | --- | --- |
| **Primer Name** | **Forward Sequence** | **Reverse Sequence** |
| CRE1 | GGTTGTAAATGCCAGCAGGT | GTGAGTGCAGCGGGATAAAT |
| CRE2 | TTCCTTAGGCACTCTTCAACAA | ACCACTCACATTGCCAAACC |
| CRE3 | TAAATGGGCCCTGTTGTGTT | ACATGATGTGCTGCAGAGGA |

| **Table S7: Primer used to amplify riboprobe templates and CRE1-3** | | | |
| --- | --- | --- | --- |
| Product | Forward primer (5’-3’) | Reverse Primer (5’-3’) | Product size (bp) |
| eGFP probe | CACATGAAGCAGCACGACTT | TAAGCTTTAATACGACTCACTATAGGGAGATGCTCAGGTAGTGGTTGTCG | 428 |
| mCherry probe | ACGGCTCCAAGGCCTACGTGA | TAAGCTTTAATACGACTCACTATAGGGAGACGTTGTGGGAGGTGATGTCCA | 430 |
| CRE3 | GCCCCAGAGGGCTCCTGTG | TGCTCACAGGAGAGACTATTGC | 1741 |
| CRE2 | CCTTTGGGCTTCTTGAACATG | TTGGTTTCCAGGACCCCTGGA | 1029 |
| CRE1 | CCTTCGTCTGTGTGTTTGTG | TGTTTCAAAACCTAAGTTGGCCT | 1195 |

| Table S8: Highly Conserved Non-Coding Elements in the *SABT2-PLCL1* Gene Desert | | | | | |
| --- | --- | --- | --- | --- | --- |
| **Chr** | **Start** | **Stop** | **Length** | **Percent identity** | **HCNE** |
| chr2 | 199180670 | 199180894 | 225 | 0.796 |  |
| chr2 | 199183363 | 199183700 | 338 | 0.811 |  |
| chr2 | 199188906 | 199189193 | 288 | 0.833 |  |
| chr2 | 199202177 | 199203109 | 933 | 0.912 | CRE1 |
| chr2 | 199205708 | 199205890 | 183 | 0.792 |  |
| chr2 | 199210735 | 199210884 | 150 | 0.8 |  |
| chr2 | 199216007 | 199216479 | 473 | 0.869 | CRE2 |
| chr2 | 199227162 | 199228070 | 909 | 0.945 | CRE3 |
| chr2 | 199276410 | 199277067 | 658 | 0.856 |  |
| chr2 | 199300628 | 199300858 | 231 | 0.784 |  |
| chr2 | 199343415 | 199343777 | 363 | 0.862 |  |
| chr2 | 199397621 | 199398011 | 391 | 0.839 |  |
| chr2 | 199398068 | 199398242 | 175 | 0.811 |  |
| chr2 | 199409513 | 199409805 | 293 | 0.826 |  |
| chr2 | 199452122 | 199452501 | 380 | 0.853 |  |
| chr2 | 199456373 | 199456676 | 304 | 0.803 |  |
| chr2 | 199478306 | 199478471 | 166 | 0.777 |  |
| chr2 | 199478488 | 199478652 | 165 | 0.812 |  |
| chr2 | 199505353 | 199505603 | 251 | 0.849 |  |
| chr2 | 199515393 | 199515568 | 176 | 0.795 |  |
| chr2 | 199522989 | 199523567 | 579 | 0.888 |  |
| chr2 | 199527662 | 199527884 | 223 | 0.785 |  |
| chr2 | 199538132 | 199538290 | 159 | 0.805 |  |
| chr2 | 199557733 | 199557985 | 253 | 0.798 |  |
| chr2 | 199558053 | 199558291 | 239 | 0.778 |  |
| chr2 | 199560563 | 199560836 | 274 | 0.792 |  |
| chr2 | 199571598 | 199571752 | 155 | 0.813 |  |
| chr2 | 199600305 | 199600477 | 173 | 0.798 |  |
| chr2 | 199615672 | 199615863 | 192 | 0.781 |  |
| chr2 | 199617822 | 199618104 | 283 | 0.827 |  |
| chr2 | 199626648 | 199627189 | 542 | 0.83 |  |
| chr2 | 199730965 | 199731615 | 651 | 0.833 |  |
| chr2 | 199738738 | 199738891 | 154 | 0.799 |  |
| chr2 | 199832605 | 199833334 | 730 | 0.877 |  |
| chr2 | 199936338 | 199936518 | 181 | 0.801 |  |
| chr2 | 199961165 | 199961392 | 228 | 0.864 |  |
| chr2 | 200066451 | 200066611 | 161 | 0.807 |  |
| chr2 | 200067691 | 200067885 | 195 | 0.795 |  |
| chr2 | 200071354 | 200071521 | 168 | 0.786 |  |
| chr2 | 200075656 | 200075966 | 311 | 0.817 |  |
| chr2 | 200133694 | 200133976 | 283 | 0.799 |  |

| **Table S9 Consistence of reporter transgenic activity in stable zebrafish lines** | | | |
| --- | --- | --- | --- |
| **Putative enhancer *** | **Reporter used in transgenic assay ^#^** | **Number of stable transgenic lines ^#^analysed** | **Sites of reporter expression driven by the element** |
| CRE1 | eGFP | 6 | Weak ubiquitous (2/6)  Heart (2/6)  Yolk sac (1/6)  Weak lens (1/6) |
| CRE2-WT | mCherry | 4 | **Ethmoid plate (4/4)**  Pharyngeal arch (2/4)  Brain (1/4)  Otic vesicle (1/4)  Olfactory placode (1/4) |
| CRE2-SOX9m | eGFP | 4 | **Ethmoid plate (0/4)**  Olfactory placode (1/4)  Lens (1/4)  Brain (1/4)  Otic vesicle (1/4) |
| CRE3 | eGFP | 4 | **Olfactory placode (4/4)**  Pectoral fin (1/4)  Heart (1/4) |

* Species of origin: *Homo sapiens*

# Model organism for reporter transgenic assay: *Danio rerio*
